# Public Knowledge, Attitudes, and Practices Regarding Skin Cancer, Risk Factors, and Preventive Measures in Saudi Arabia: A Cross-Sectional Study

**DOI:** 10.3390/jcm15218570

**Published:** 2026-11-03

**Authors:** Munerah Hamed

**Affiliations:** Department of Pathology, College of Medicine, Umm Al-Qura University, Makkah 21955, Saudi Arabia; mhhamed@uqu.edu.sa

**Keywords:** skin cancer, public knowledge, public attitude, preventive practices, Saudi Arabia, ultraviolet

## Abstract

**Background/Objectives**: Skin cancer is a largely preventable disease associated with ultraviolet radiation exposure that represents an important public health concern in Saudi Arabia. In the context of ongoing social and lifestyle changes, this study aimed to assess knowledge, attitudes, and practices regarding skin cancer and examine their associations with sociodemographic characteristics among surveyed adults residing in Saudi Arabia. **Methods**: This cross-sectional study used a self-administered online questionnaire distributed through social media platforms. A total of 451 participants aged ≥18 years residing in Saudi Arabia were included. Data comprised sociodemographic characteristics and knowledge, attitudes, and practices regarding skin cancer. Descriptive statistics are presented as mean ± standard deviation (SD) and median (interquartile range [IQR]) for continuous variables and frequencies and percentages for categorical variables. Non-parametric and multiple linear regression analyses were used to assess associations between sociodemographic characteristics and knowledge, attitude, and practice scores. **Results**: The median knowledge, attitude, and practice scores were 13.0/26 (IQR 10.0–16.0), 6.0/12 (IQR 4.0–7.0), and 5.0/16 (IQR 4.0–6.0), respectively. Sex, education level, and employment status were significantly associated with knowledge scores. Age, sex, education level, employment status, and income were associated with attitude scores. Sex, employment status, and income were associated with practice scores. **Conclusions**: This study identified differences in skin cancer knowledge, attitudes, and preventive practices among adults in Saudi Arabia, highlighting opportunities to strengthen preventive behaviors. Targeted public health interventions may help promote skin cancer awareness and prevention.

## 1. Introduction

Non-melanoma skin cancers represent approximately 20% of all cancers, and their incidence is consistently increasing [1,2]. Accurate determination of the incidence of skin cancer remains challenging owing to underreporting [3]. Skin cancer primarily comprises malignant melanoma and non-melanoma skin cancers, with each type leading to distinctly different clinical outcomes [4]. Malignant melanoma accounts for only 5% of all skin cancers, and its stage at diagnosis plays a critical role in determining patient prognosis [5]. Although melanoma and non-melanoma skin cancers are regarded as multifactorial diseases, prolonged and unprotected exposure to natural ultraviolet (UV) radiation or UV lamps is the primary cause and risk factor for skin cancer [1]. Skin cancer development is influenced by multiple factors, including environmental exposures and individual susceptibility. Beyond UV exposure, genetic predisposition also contributes to melanoma risk, as demonstrated by Ciccarese et al., who identified distinct clinical and pathological characteristics among patients carrying the *MITF* p.E318K variant. These findings highlight the multifactorial nature of skin cancer and the importance of preventive strategies and early detection [6]. At the molecular level, cumulative UV exposure contributes to carcinogenesis by inducing mutations in key driver genes, such as *TP53* and *NOTCH1*, leading to genomic instability and malignant transformation [7]. Notably, non-melanoma skin cancer commonly develops in skin areas that are frequently exposed to UV radiation, such as the head, neck, back of the hands, and trunk, especially among individuals with outdoor occupations, such as farmers, sailors, and fishermen [1,8].

Cancer represents a growing public health burden in Saudi Arabia, with 25,217 newly diagnosed cases reported to the Saudi Cancer Registry in 2023 among adults aged ≥ 14 years. Although skin cancers, including melanoma and other skin malignancies, account for a relatively small proportion of all cancer cases [9], they remain clinically significant because of their preventable nature. The presence of melanoma and non-melanoma skin cancers in national statistics highlights their ongoing incidence in the Saudi population [9]. Accordingly, increasing public awareness and promoting protective behaviors against UV exposure are essential for reducing the future burden of skin cancer. The Vision 2030 coastal and urban developments of Saudi Arabia, including the expansion of beaches and recreational parks, are expected to increase population-level engagement in outdoor environments [10].

Concurrently, the beauty sector is rapidly expanding, with commercial registrations for beauty centers increasing by 83%, reflecting a marked shift toward cosmetic practices and aesthetic services [11]. Within this context, the growing popularity of gel nail products requiring UV-emitting curing devices raises questions about potential cumulative exposure and its implications for skin health. Experimental studies have demonstrated that UV nail dryers can induce oxidative stress, DNA damage, mitochondrial dysfunction, and persistent mutations in mammalian cells, as well as cytotoxic effects in human skin cells [12,13]. A case report has described squamous cell carcinoma on the female hands with long-term or frequent exposure to UV nail lamps [14]. This concern is biologically plausible within the broader dermatology literature because ultraviolet radiation is a recognized human carcinogen, and the related review discussing tanning beds and nail lamps places nail-lamp exposure within this wider UV-carcinogenesis framework [15,16]. Moreover, evidence from large-scale meta-analyses has concluded that artificial UV sources, such as indoor tanning, are established carcinogens associated with a significantly increased risk of squamous and basal cell carcinomas [17]. Additionally, cumulative UV exposure from occupational and recreational sources has been linked to actinic keratosis and non-melanoma skin cancer [18]. Population-based studies have reported rising incidences of basal and squamous cell carcinomas in Belgium, partly attributed to increased sun exposure, behavioral changes, aging, and improved cancer registration [19], whereas Australian data indicate that solar keratoses may serve as a sensitive marker of sun exposure in sun-exposed areas [20]. Therefore, the concurrent increase in environmental and artificial sources of UV exposure, including outdoor recreational activities and cosmetic procedures, represents an emerging public health concern. Given the documented biological effects of UV radiation and increasing exposure through lifestyle- and beauty-related practices, there is a critical need to evaluate public awareness and behaviors related to skin cancer prevention.

This study addresses a current public health gap by examining knowledge, attitudes, and preventive practices regarding skin cancer among surveyed adults residing in Saudi Arabia during a period of changing UV-exposure opportunities. While previous Saudi studies have often focused on selected regional samples, students, or female university populations, less is known about whether awareness translates into preventive behavior in the context of expanding outdoor recreational spaces, increased mobility, and growing cosmetic UV exposure [10]. Given the preventable nature of skin cancer, understanding these gaps is important for guiding targeted public health interventions and promoting protective behaviors in everyday settings.

## 2. Materials and Methods

### 2.1. Study Population

This study was approved by the Biomedical Research Ethics Committee at Umm Al-Qura University (No. HAPO-02-K-012-2024-12-2354) and was conducted in accordance with the principles of the Declaration of Helsinki. All participants provided informed consent before participating in the study, and all data were anonymized.

This cross-sectional study included 451 participants aged ≥18 years residing in Saudi Arabia. The minimum required sample size was 206 participants, calculated using Epi Info (version 5.5.11; Centers for Disease Control and Prevention, Atlanta, GA, USA) at the 95% confidence level, assuming a preventive practice prevalence of 50% among participants with adequate knowledge and 30% among those with limited knowledge.

### 2.2. Data Collection

Data were collected between February and May 2025 using an online survey distributed through WhatsApp. Snowball sampling was used. Of a total of 611 participants, 451 were included in the final analysis, while 160 responses were excluded. Among those excluded, five declined consent or were below 18 years of age, while the remaining 155 had contradictory answer selections, inconsistent responses to related practice questions, or male responses to female-specific questions. Sociodemographic data included coastal region, age group, sex, marital status, education level, employment status, and monthly income (Saudi Riyal [SR] categories). The questionnaire was adapted from a previously published Saudi study of medical students [21]. The questionnaire was translated into Arabic independently by two researchers and subsequently reviewed by two experts in epidemiology for content validity. It was pilot-tested among 30 participants to assess clarity and comprehensibility; subsequently, overlapping response options were removed and “I don’t know” responses were standardized. The internal consistency of the knowledge and attitude scales was high (Cronbach’s alpha = 0.87 and 0.74, respectively). Internal consistency was not reported for the practice items because they assessed distinct aspects of skin cancer prevention behavior. Accordingly, the practice score was treated as an exploratory composite index.

### 2.3. Assessment of Knowledge, Attitudes, and Practices Toward Skin Cancer

A structured questionnaire was used to assess knowledge, attitudes, and practices related to skin cancer among the public in Saudi Arabia. Knowledge was evaluated using 26 items on skin cancer. Responses were coded as correct (1) or incorrect (0), yielding a total score ranging from 0 to 26, with higher scores indicating greater knowledge. Attitudes toward skin cancer were assessed using six items addressing perceptions of skin cancer. Responses were recorded on a five-point Likert scale and coded as follows: strongly agree = 2, agree = 1, and neutral/disagree/strongly disagree = 0, resulting in a total score ranging from 0 to 12. Higher scores indicated favorable attitudes. Preventive practices were assessed using eight items for the overall sample (score range, 0–16) and 10 items for females (2 additional items related to UV nail lamps and UV nail lamp glove use) (score range, 0–20). Positive behaviors were scored as always = 2, often = 1, and sometimes/rarely/never = 0, whereas negative behaviors were reverse-coded. Higher scores indicated better preventive practices (Table 1).

### 2.4. Statistical Analysis

Continuous variables are summarized as mean ± standard deviation (SD) and median (interquartile range), whereas categorical variables are summarized as frequencies and percentages. The normality of knowledge, attitudes, and practices scores was assessed using the Shapiro–Wilk test and visual inspection of distributions. Because the knowledge, attitudes, and practices scores were non-normally distributed, group differences were assessed using the Mann–Whitney U test for two-group comparisons and the Kruskal–Wallis test for comparisons involving more than two groups, with Bonferroni correction for post hoc comparisons. Effect sizes were calculated as r = |Z|/√N for Mann–Whitney U tests and H-based eta-squared (η^2^H) = max [0, (H − k + 1)/(N − k)] for Kruskal–Wallis tests, where N is the sample size and k is the number of groups.

Spearman’s correlation was used to assess relationships among knowledge, attitude, and practice scores. Multiple linear regression analyses were performed to examine associations between sociodemographic characteristics and knowledge, attitude, and practice scores after adjustment for other covariates. An additional hierarchical linear regression was performed to examine whether knowledge and attitude scores were independently associated with preventive practice scores after adjustment for sociodemographic characteristics. Sociodemographic variables were entered simultaneously in the first block, followed by knowledge and attitude scores in the second block. Categorical independent variables were dummy-coded, with non-coastal region, male sex, unmarried status, unemployed status, university education, monthly income < SAR 5000, and age 18–30 years used as reference categories. Separate analyses were conducted for the overall sample (*n* = 451) and female participants (*n* = 218), with sex excluded from the latter models.

Regression assumptions were assessed by examining residual plots, histograms, normal probability plots of standardized residuals, and variance inflation factor values to evaluate linearity, homoscedasticity, normality of residuals, and multicollinearity. Cronbach’s alpha was used to assess scale reliability. Exact *p*-values and 95% confidence intervals (CIs) were reported where applicable, and statistical significance was set at *p* < 0.05. Statistical analyses were performed using IBM SPSS Statistics (version 25.0; IBM Corp., Armonk, NY, USA).

## 3. Results

### 3.1. Sociodemographic Characteristics

A total of 451 participants were included in the study. Most participants were from non-coastal regions (64.5%; *n* = 291). The largest age group was 31–50 years (48.3%, *n* = 218), followed by 18–30 years (36.1%, *n* = 163). Males accounted for 51.7% (*n* = 233) of the sample. Most participants were married (69.6%, *n* = 314), and 57.9% (*n* = 261) had a university degree. In total, 25.1% (*n* = 113) of the participants were employed in the healthcare sector. The most common income categories were SR 10,001–15,000 and SR 15,001–20,000 (28.4%, *n* = 128 each; Table 2).

### 3.2. Associations Between Knowledge, Attitudes, and Practices Scores and Sociodemographic Characteristics

The mean knowledge score was 13.04 ± 4.50 (median, 13.00 [10.00–16.00]). Knowledge was significantly associated with sex (*p* = 0.003), education level (*p* < 0.001), and employment status (*p* = 0.002), with higher scores observed among females and participants with higher levels of education. Students (*p* = 0.010) and participants employed in the healthcare sector (*p* = 0.003) had higher knowledge scores than unemployed participants.

The mean attitude score was 6.06 ± 2.63 (median, 6.00 [4.00–7.00]). Attitudes were significantly associated with region (*p* = 0.042), age (*p* < 0.001), sex (*p* < 0.001), education level (*p* < 0.001), employment status (*p* < 0.001), and income (*p* < 0.001). Higher attitude scores were observed among older participants, females, and those with higher levels of education. Participants employed in the healthcare sector had lower attitude scores than those who were unemployed or employed in non-healthcare sectors. Participants in the lowest income group had higher attitude scores than those in the higher income groups.

The mean practice score was 4.92 ± 2.17 (median, 5.00 [4.00–6.00]). Practice was significantly associated with sex (*p* = 0.005) and income (*p* < 0.001). Females had higher practice scores than males. Participants with lower income (≤SR 5000) had higher practice scores than those in higher income groups. Overall, the observed group differences were generally small in magnitude. Effect-size estimates for group comparisons and detailed results are presented in Table 3.

Among females, knowledge scores were significantly associated with education level (*p* < 0.004) and income (*p* = 0.007). Attitude scores were significantly associated with region (*p* = 0.042), age (*p* < 0.001), education level (*p* < 0.004), employment status (*p* < 0.004), and income (*p* < 0.004). Finally, practice scores were significantly associated with age (*p* = 0.010), education level (*p* = 0.032), employment status (*p* = 0.005), and income (*p* < 0.001). Detailed results are presented in Table S1. In the subgroup analysis of responses from female participants (*n* = 218), effect sizes were generally small, with the largest estimates observed for age in relation to attitude scores (η^2^H = 0.065) and monthly income in relation to practice scores (η^2^H = 0.075). Spearman’s correlation analysis revealed weak positive correlations between knowledge and attitudes (ρ = 0.262, *p* < 0.001), between knowledge and preventive practices (ρ = 0.106, *p* = 0.024), and between attitudes and preventive practices (ρ = 0.257, *p* < 0.001) regarding skin cancer. All correlations were significant and were based on 451 participants.

### 3.3. Regression Analysis

Multiple linear regression analyses showed that the overall models were significant for knowledge (F(15, 435) = 4.168, *p* < 0.001, R^2^ = 0.126), attitudes (F(15, 435) = 6.314, *p* < 0.001, R^2^ = 0.179), and practices (F(15, 435) = 2.866, *p* < 0.001, R^2^ = 0.090) regarding skin cancer. Knowledge scores were significantly higher among female participants (B = 1.624, *p* < 0.001), students (B = 3.055, *p* < 0.001), healthcare employees (B = 2.693, *p* < 0.001), and participants with higher education (B = 1.846, *p* < 0.004), compared with their respective reference categories (Table 4). Attitude scores were significantly higher among females (B = 0.709, *p* = 0.003), participants aged 31–50 years (B = 0.972, *p* < 0.001), and those with higher education (B = 0.769, *p* = 0.036), whereas healthcare employees (B = −0.866, *p* = 0.023) and those with monthly incomes of SAR 10,001–15,000 (B = −1.667, *p* < 0.004) and SAR 15,001–20,000 (B = −1.839, *p* = 0.002) demonstrated significantly lower attitude scores. Practice scores were significantly higher among female participants (B = 0.589, *p* = 0.005), whereas non-healthcare employees (B = −0.673, *p* = 0.028) and participants in all income categories above SAR 5000 exhibited significantly lower scores (all *p* < 0.05) (Table 4). In the additional adjusted model, attitude scores were positively associated with practice scores (B = 0.233, 95% CI: 0.148–0.317, *p* < 0.001), whereas knowledge scores were not significantly associated (B = 0.046, 95% CI: −0.002–0.093, *p* = 0.060). Adding both scores increased R^2^ from 0.090 to 0.180 (ΔR^2^ = 0.090, *p* < 0.001).

In the subgroup analysis of responses from female participants, multiple linear regression models were statistically significant for knowledge (F(14, 203) = 2.689, *p* < 0.001, R^2^ = 0.156), attitudes (F(14, 203) = 3.280, *p* < 0.001, R^2^ = 0.184), and practices (F(14, 203) = 2.413, *p* < 0.004, R^2^ = 0.143) regarding skin cancer. Higher education was significantly associated with increased knowledge scores (B = 2.533, *p* = 0.012) (Table S2). Attitude scores were significantly higher among female participants aged 31–50 years (B = 1.271, *p* = 0.008) and lower among healthcare employees (B = −1.261, *p* = 0.031). Practice scores were significantly lower among non-healthcare employees (B = −1.380, *p* = 0.009), healthcare employees (B = −1.487, *p* = 0.009), and those with monthly incomes of SAR 5001–10,000 (B = −2.007, *p* = 0.019) (Table S2). The full regression coefficients are presented in Table 4 and Table S2.

## 4. Discussion

Skin cancer remains an important public health concern in Saudi Arabia despite its relatively low incidence compared with other malignancies, largely because of its preventable nature [9]. Notably, UV radiation from sunlight and artificial sources is a well-established contributor to skin cancer risk [12,17]. Recent societal and lifestyle changes in Saudi Arabia, including expanded recreational spaces, increased mobility, and growth in cosmetic services, provide an important public health context for strengthening skin cancer awareness and prevention efforts [10]. Accordingly, this study focused on assessing public knowledge, attitudes, and practices regarding skin cancer prevention and examining their independent associations with sociodemographic characteristics.

The findings of this study showed median knowledge, attitude, and preventive practice scores of 13.0/26 (IQR 10.0–16.0), 6.0/12 (IQR 4.0–7.0), and 5.0/16 (IQR 4.0–6.0), respectively. Spearman’s correlation analysis demonstrated weak positive correlations between knowledge and attitudes, between knowledge and preventive practices, and between attitudes and preventive practices regarding skin cancer. Although all three correlations were statistically significant, their magnitudes were relatively small. In the adjusted regression analysis, knowledge scores were not significantly associated with preventive practice scores after controlling for attitude scores and sociodemographic characteristics, whereas the attitude scores remained positively associated with preventive practice scores.

The results of this study showed that several sociodemographic factors were significantly associated with participants’ knowledge, attitudes, and preventive practices regarding skin cancer. In the total sample, female participants, students, healthcare employees, and those with postgraduate education demonstrated significantly higher knowledge scores compared with their respective reference groups. Female participants, participants aged 31–50 years, and those with postgraduate education reported more favorable attitudes, whereas participants employed in the healthcare sector and participants in certain higher-income categories demonstrated lower attitude scores. For preventive practices, female participants demonstrated higher scores, whereas participants employed in the non-healthcare sector and those with monthly incomes exceeding SAR 5000 demonstrated lower scores compared with their respective reference groups. Overall, the predominantly small effect sizes observed in both the total sample and subgroup of female participants suggest that sociodemographic differences may have limited practical significance. Furthermore, although the regression models explained a greater proportion of outcome variability, their explanatory power remained modest (R^2^ = 0.126, 0.179, and 0.090) for knowledge, attitudes, and practices, respectively, suggesting that additional factors may contribute to variation in these outcomes. In the subgroup analysis of responses from female participants, postgraduate education was associated with higher knowledge scores, whereas participants aged 31–50 years demonstrated more favorable attitudes, and participants employed in the healthcare sector demonstrated lower attitude scores. Regarding preventive practices specific to female individuals, employment in healthcare or non-healthcare sectors and monthly income of SAR 5001–10,000 were significantly associated with lower practice scores compared with their respective reference groups. The adjusted regression coefficients indicated varying magnitudes of association across sociodemographic characteristics. For example, female sex was associated with higher knowledge scores (B = 1.624, 95% CI: 0.787–2.461), while healthcare employment was associated with lower attitude scores (B = −0.866, 95% CI: −1.614 to −0.119), compared with their respective reference groups.

Many studies have identified significant associations between sociodemographic factors and knowledge regarding sun exposure and skin cancer [22,23]. For example, a study conducted among female university students in Abha, Saudi Arabia, demonstrated that academic level was significantly associated with knowledge, with higher-year students showing better awareness of sun-related risks. Moreover, knowledge was positively correlated with attitudes [22]. Accordingly, our study showed that participants with postgraduate education exhibited significantly higher knowledge scores than did those with university education, which may be attributed to greater academic exposure. However, improved knowledge did not consistently translate into better practices, indicating that other factors, including cultural beliefs and personal behaviors, may influence the adoption of protective measures.

Several studies conducted in Saudi Arabia have reported varying levels of knowledge, attitudes, and practices regarding skin cancer and sun protection. For instance, a study of medical students reported relatively high levels of knowledge and awareness, likely because of their medical background and exposure to health education [21]. In line with these findings, our study showed that participants employed in the healthcare sector had significantly higher knowledge scores than did unemployed participants. However, despite this higher level of knowledge, participants employed in the healthcare sector demonstrated significantly lower attitude scores in the overall sample, whereas female participants employed in the healthcare sector also exhibited significantly lower preventive practice scores compared with unemployed participants. The observed differences between awareness and preventive practice scores suggest that greater knowledge does not necessarily correspond to greater engagement in preventive behaviors. Occupational factors, such as demanding work schedules and predominantly indoor environments, may partly contribute to these differences; however, these factors were not directly assessed in this study. A similar pattern was reported in a recent study from Iran, in which general practitioners had greater knowledge than medical students but were less likely to demonstrate good practice; this finding was attributed to declining personal adherence to sun protection as clinicians progressed from training to clinical practice [24]. These findings suggest that knowledge alone may not fully explain preventive behaviors, which may also be associated with attitudes and other unmeasured factors. Future research should examine whether attitudes and workplace-related factors are associated with preventive practices among healthcare employees.

Previous studies in Saudi Arabia have reported relatively high awareness of UV exposure and its harmful effects; however, knowledge regarding specific preventive measures remains inconsistent [23]. For example, a study conducted in Jazan demonstrated better public awareness of proper sunscreen use, including reapplication practices [25]. Conversely, our findings showed gaps in knowledge regarding preventive behaviors and sunscreen use, with only 47.0% of participants identifying sunscreen as a protective measure against skin cancer. These differences may reflect variations in study populations, health awareness, and access to educational resources across different regions; however, these potential explanations were not directly evaluated in this study. The same study conducted in Jazan, a coastal region of Saudi Arabia, reported better preventive practices against sun exposure [25]. Nevertheless, our findings showed no significant association between coastal residence and preventive practices scores, after adjustment for other sociodemographic factors. This discrepancy indicates that residing in a coastal region alone may not necessarily translate into improved protective behavior. Preventive practices may also be considered in the context of recent social changes in Saudi Arabia. As women were permitted to drive in Saudi Arabia in 2018, increased mobility may have created additional opportunities for UV exposure, highlighting the potential relevance of driving-related sun protection. In our study, only 44.8% of participants reported often or always wearing gloves while driving (Table 1). Given the potential risks associated with prolonged UV exposure, these findings highlight the importance of considering everyday activities, including driving, in skin cancer prevention initiatives.

This study adds to the existing literature by examining skin cancer knowledge, attitudes, and practices in Saudi Arabia during a period of social and lifestyle change, including the expansion of public recreational spaces, increased mobility, and growth in cosmetic services. However, this study had some limitations. First, its cross-sectional design limits the ability to establish causal relationships among knowledge, attitudes, and practices or to assess changes over time. Second, the generalizability of the findings may be limited with a convenience sampling approach, which may not fully represent all segments of the population. Furthermore, the use of an online survey may have introduced selection bias by potentially overrepresenting individuals with higher education levels, greater health awareness, or better access to digital platforms. Furthermore, the exclusion of non-applicable or inconsistent responses, although necessary to ensure data quality, may have introduced selection bias. Additionally, occupational schedules, working environments, driving duration, and outdoor activity duration were not directly measured. UV nail-lamp use and tanning practices were self-reported, and no objective measurements of UV dose or cumulative exposure were obtained, limiting interpretation of the proposed exposure-related explanations. Future studies should include these measures to better evaluate how lifestyle-related exposures influence skin cancer prevention behaviors.

## 5. Conclusions

Our findings add updated Saudi evidence by showing that sex, education level, age, employment status, and income were associated with skin-cancer awareness and prevention behaviors. Although knowledge and attitudes were positively correlated with preventive practices, only attitudes remained significantly associated with preventive practices after adjustment for sociodemographic characteristics. These findings highlight the potential value of context-specific skin cancer prevention strategies. Future studies should directly assess UV exposure during outdoor activities, driving, and cosmetic procedures to inform the development of targeted interventions. To develop a more comprehensive understanding of these behaviors, future studies should directly measure outdoor sun exposure and include key potential confounders, such as Fitzpatrick skin type, occupational sun exposure, family skin-cancer history, sunscreen affordability, urban/rural residence, and prior dermatology consultation.

## Figures and Tables

**Table 1:** Knowledge, attitudes, and practices regarding skin cancer among study participants in Saudi Arabia (*n* = 451).

No.	Variable	*n*	%
Knowledge of skin cancer			
	Symptoms of skin cancer		
1	New or changing moles (size, color, texture, edges). Yes	211	46.8
2	Superficial lesions (flat, scaly; usually red or pink). Yes	192	42.6
3	Nodular (pearly/waxy, visible blood vessels; may ulcerate/bleed). Yes	166	36.8
4	Abnormal skin texture (firm and skin-colored). Yes	188	41.7
5	Darkly pigmented, like a mole; color varies from brown to black. Yes	102	22.6
	IDK	89	19.7
	Damage caused by excessive sun exposure		
6	Skin burns. Yes	209	46.3
7	Wrinkles. Yes	220	48.8
8	Skin aging. Yes	205	45.5
9	Hyperpigmentation. Yes	196	43.5
10	Skin cancer. Yes	230	51.0
	Has no effect/IDK	41	9.1
	Risk factors of skin cancer		
11	UV exposure. Yes	195	43.2
12	Fair skin. Yes	162	35.9
13	History. Yes	213	47.2
14	Immunosuppression. Yes	155	34.4
	IDK	50	11.1
	Sources of UV		
15	Sun. Yes	184	40.8
16	Tanning beds. Yes	309	68.5
17	Lamps used to dry gel nail polish. Yes	209	46.3
	IDK	47	10.4
	Highest UV level during the day		
18	10 am to 4 pm. Yes (correct answer)	322	71.4
19	8 am to 10 am. No	430	95.3
20	4 pm to sunset. No	395	87.6
21	All daytime. No	420	93.1
	IDK	20	4.4
	Preventive measures to avoid skin cancer		
22	Sunscreen. Yes	212	47.0
23	Avoiding UV peak hours. Yes	263	58.3
24	Wearing gloves while driving. Yes	188	41.7
25	Wearing gloves when using a UV lamp for gel polish. Yes	153	33.9
26	Doing regular skin examinations. Yes	150	33.3
	IDK	21	4.7
Attitude towards skin cancer			
1	Skin cancer is a serious problem		
	Strongly agree/agree	339	75.2
	Neutral	58	12.9
	Strongly disagree/disagree	54	12.0
2	UV can cause skin cancer		
	Strongly agree/agree	275	61.0
	Neutral	98	21.7
	Strongly disagree/disagree	78	17.3
3	Need sunscreen even on non-sunny days		
	Strongly agree/agree	286	63.4
	Neutral	92	20.4
	Strongly disagree/disagree	73	16.2
4	Need to visit a doctor for suspicious skin changes		
	Strongly agree/agree	318	70.5
	Neutral	67	14.9
	Strongly disagree/disagree	66	14.6
5	Need regular skin examination if there is a history of skin cancer		
	Strongly agree/agree	312	69.2
	Neutral	70	15.5
	Strongly disagree/disagree	69	15.3
6	Need protection to avoid skin cancer		
	Strongly agree/agree	328	72.7
	Neutral	80	17.7
	Strongly disagree/disagree	43	9.5
Practices related to skin cancer			
1	I use sunscreen		
	Never/rarely	153	33.9
	Sometimes	81	18.0
	Often/always	217	48.1
2	I use gloves while driving		
	Never/rarely	194	43.0
	Sometimes	55	12.2
	Often/always	202	44.8
3	I use a hat/head cover		
	Never/rarely	175	38.8
	Sometimes	101	22.4
	Often/always	175	38.8
4	I undergo regular skin examination		
	Never/rarely	198	43.9
	Sometimes	62	13.7
	Often/always	191	42.4
5	I see a doctor if I have suspicious skin changes		
	Never/rarely	92	20.4
	Sometimes	92	20.4
	Often/always	267	59.2
6	I wear gloves when using a UV lamp to dry gel polish *		
	Never/rarely	112	51.4
	Sometimes	20	9.2
	Often/always	86	39.4
7	I use gel nail polish that requires a UV lamp to dry *		
	Never/rarely	75	34.4
	Sometimes	38	17.4
	Often/always	105	48.2
8	I tan at the beach		
	Never/rarely	232	51.4
	Sometimes	72	16.0
	Often/always	147	32.6
9	I tan at the beach between 10 am and 4 pm		
	Never/rarely	182	40.4
	Sometimes	96	21.3
	Often/always	173	38.4
10	I use UV tanning beds		
	Never/rarely	201	44.6
	Sometimes	53	11.8
	Often/always	197	43.7

* *n* = 218 (females only). IDK, I do not know; UV, ultraviolet.

**Table 2:** Sociodemographic characteristics of the study participants (*n* = 451).

Characteristics	*n*	%
Coastal region		
Non-coastal region	291	64.5
Coastal region	160	35.5
Age (years)		
18–30	163	36.1
31–50	218	48.3
>50	70	15.5
Sex		
Male	233	51.7
Female	218	48.3
Marital status		
Not married	137	30.4
Married	314	69.6
Education level		
<High school	12	2.7
High school/diploma	101	22.4
University	261	57.9
Higher education	77	17.1
Employment status		
Unemployed	89	19.7
Student	59	13.1
Employed in the non-healthcare sector	190	42.1
Employed in the healthcare sector	113	25.1
Monthly income		
≤SR 5000	23	5.1
SR 5001–10,000	67	14.9
SR 10,001–15,000	128	28.4
SR 15,001–20,000	128	28.4
>SR 20,000	105	23.3

SR: Saudi Riyal.

**Table 3:** Knowledge, attitude, and practice scores based on participant sociodemographic characteristics.

Variable	Category	SC Knowledge Score out of 26 Mean ± SD Median (IQR)	SC Attitude Score out of 12 Mean ± SD Median (IQR)	SC Practice Score out of 16 Mean ± SD Median (IQR)
Region	Non-Coastal	12.82 ± 4.26 13.00 (10.00–16.00)	5.83 ± 2.50 5.00 (4.00–7.00)	4.99 ± 2.17 5.00 (4.00–6.00)
	Coastal	13.43 ± 4.90 13.00 (10.00–16.00)	6.48 ± 2.81 6.00 (4.00–8.00)	4.78 ± 2.17 5.00 (3.00–6.00)
	*p*-value	0.292	0.042 *	0.311
	Effect size	0.060	0.114	0.057
Age in years	18–30	13.07 ± 3.64 13.00 (11.00–16.00)	5.19 ± 1.84 5.00 (4.00–6.00)	4.67 ± 2.07 5.00 (3.00–6.00)
	31–50	13.10 ± 4.59 13.00 (10.00–16.00)	6.61 ± 2.93 6.00 (5.00–9.00)	5.10 ± 2.25 5.00 (4.00–6.00)
	>50	12.76 ± 5.89 13.50 (8.75–16.25)	6.39 ± 2.71 6.00 (4.00–8.00)	4.93 ± 2.11 5.00 (3.00–6.00)
	*p*-value	0.837	<0.001 *	0.101
	Effect size	0.000	0.046	0.006
Gender	Male	12.31 ± 4.10 13.00 (10.00–15.00)	5.58 ± 2.22 5.00 (4.00–6.50)	4.55 ± 1.76 4.00 (3.00–6.00)
	Female	13.81 ± 4.79 14.00 (10.75–17.00)	6.57 ± 2.93 6.00 (4.00–8.25)	5.31 ± 2.48 5.00 (4.00–6.00)
	*p*-value	0.003 *	<0.001 *	0.005 *
	Effect size	0.164	0.191	0.152
Marital status	Not married	13.39 ± 4.48 13.00 (11.00–16.00)	5.99 ± 2.52 6.00 (4.00–7.00)	4.75 ± 2.37 4.00 (3.00–6.00)
	Married	12.88 ± 4.51 13.00 (10.00–16.00)	6.09 ± 2.68 6.00 (4.00–7.00)	4.99 ± 2.08 5.00 (4.00–6.00)
	*p*-value	0.112	0.775	0.094
	Effect size	0.094	0.017	0.098
Education level	<High school	11.75 ± 4.85 13.50 (7.00–15.00)	6.25 ± 2.34 6.00 (4.25–8.50)	5.75 ± 2.90 5.00 (4.00–6.75)
	High school/diploma	12.82 ± 3.73 13.00 (11.00–15.00)	5.40 ± 2.13 5.00 (4.00–6.50)	4.52 ± 1.96 5.00 (3.00–5.50)
	University	12.60 ± 4.56 12.00 (10.00–15.00)	5.97 ± 2.62 6.00 (4.00–7.00)	4.93 ± 2.20 5.00 (4.00–6.00)
	Higher education	15.00 ± 4.74 14.00 (12.00–18.00)	7.21 ± 2.95 7.00 (5.00–10.00)	5.27 ± 2.16 5.00 (4.00–6.00)
	*p*-value	0.001 *	<0.001 *	0.130
	Effect size	0.028	0.036	0.006
Employment status	Unemployed	11.76 ± 6.12 10.00 (7.00–15.50)	6.89 ± 2.96 7.00 (4.50–10.00)	5.51 ± 2.85 5.00 (3.50–7.00)
	Student	13.76 ± 3.80 14.00 (12.00–16.00)	5.15 ± 2.20 5.00 (4.00–6.00)	4.80 ± 2.12 5.00 (4.00–6.00)
	Employed in the non-health sector	12.91 ± 4.02 13.00 (10.00–15.00)	6.33 ± 2.75 6.00 (4.00–7.25)	4.83 ± 2.10 5.00 (3.00–6.00)
	Employed in the health sector	13.87 ± 3.87 13.00 (11.00–16.00)	5.43 ± 2.04 5.00 (4.00–6.00)	4.67 ± 1.55 5.00 (4.00–6.00)
	*p*-value	0.002 *	<0.001 *	0.194
	Effect size	0.028	0.045	0.004
Income	≤SR 5000	12.35 ± 5.40 13.00 (9.00–16.00)	7.78 ± 2.71 7.00 (5.00–10.00)	6.48 ± 2.27 6.00 (6.00–7.00)
	SR 5001–10,000	12.99 ± 4.22 13.00 (10.00–16.00)	6.18 ± 2.72 6.00 (4.00–7.00)	4.37 ± 1.98 4.00 (3.00–6.00)
	SR 10,001–15,000	12.92 ± 4.20 13.00 (10.00–16.00)	5.54 ± 2.34 5.00 (4.00–6.75)	4.94 ± 1.99 5.00 (4.00–6.00)
	SR 15,001–20,000	12.27 ± 4.01 12.00 (10.00–15.00)	5.48 ± 2.02 5.00 (4.00–6.00)	4.81 ± 2.19 5.00 (4.00–6.00)
	>SR 20,000	14.30 ± 5.16 13.00 (11.00–18.00)	6.95 ± 3.13 6.00 (5.00–10.00)	5.03 ± 2.31 5.00 (3.00–6.00)
	*p*-value	0.085	<0.001 *	0.001 *
	Effect size	0.009	0.050	0.031

* *p* < 0.05 indicates statistical significance. Data are presented as mean ± SD and median (IQR). IQR: interquartile range; SD: standard deviation; SR: Saudi Riyal; SC: skin cancer.

**Table 4:** Multivariable linear regression analyses of knowledge, attitude, and practice scores.

Independent Variable/Comparison	B	SE	*p*-Value	95% CI for B	VIF
Knowledge of participants about skin cancer					
Coastal region (coastal vs. non-coastal)	0.753	0.441	0.088	−0.113 to 1.620	1.094
Gender (female vs. male)	1.624	0.426	<0.001 *	0.787 to 2.461	1.114
Marital status (married vs. not married)	0.060	0.500	0.905	−0.923 to 1.042	1.299
Employment: student vs. unemployed	3.055	0.860	<0.001 *	1.365 to 4.745	2.068
Employment: non-healthcare vs. unemployed	1.189	0.621	0.056	−0.031 to 2.409	2.310
Employment: healthcare vs. unemployed	2.693	0.672	<0.001 *	1.373 to 4.013	2.083
Education: below high school vs. university	−0.849	1.326	0.522	−3.456 to 1.757	1.121
Education: high school/diploma vs. university	0.262	0.536	0.626	−0.793 to 1.316	1.230
Education: higher education vs. university	1.846	0.646	0.004 *	0.578 to 3.115	1.451
Income: SAR 5001–10,000 vs. <5000	−0.164	1.074	0.879	−2.274 to 1.946	3.585
Income: SAR 10,001–15,000 vs. <5000	−0.395	1.026	0.701	−2.411 to 1.622	5.263
Income: SAR 15,001–20,000 vs. <5000	−0.845	1.026	0.411	−2.862 to 1.171	5.263
Income: SAR >20,000 vs. <5000	0.733	1.065	0.492	−1.361 to 2.827	4.986
Age: 31–50 vs. 18–30	0.307	0.518	0.554	−0.711 to 1.324	1.645
Age: >50 vs. 18–30	0.268	0.736	0.716	−1.179 to 1.714	1.747
Model Fit Statistics	*n* = 451; R^2^ = 0.126; adjusted R^2^ = 0.096; F(15, 435) = 4.168; model *p* < 0.001				
Attitude of participants toward skin cancer					
Coastal region (coastal vs. non-coastal)	0.467	0.250	0.062	−0.023 to 0.958	1.094
Gender (female vs. male)	0.709	0.241	0.003 *	0.235 to 1.183	1.114
Marital status (married vs. not married)	−0.269	0.283	0.342	−0.825 to 0.287	1.299
Employment: student vs. unemployed	−0.726	0.487	0.137	−1.682 to 0.231	2.068
Employment: non-healthcare vs. unemployed	−0.458	0.351	0.193	−1.148 to 0.233	2.310
Employment: healthcare vs. unemployed	−0.866	0.380	0.023 *	−1.614 to −0.119	2.083
Education: below high school vs. university	−0.370	0.751	0.622	−1.846 to 1.105	1.121
Education: high school/diploma vs. university	−0.404	0.304	0.184	−1.001 to 0.193	1.230
Education: higher education vs. university	0.769	0.365	0.036 *	0.051 to 1.487	1.451
Income: SAR 5001–10,000 vs. <5000	−0.895	0.608	0.142	−2.089 to 0.299	3.585
Income: SAR 10,001–15,000 vs. <5000	−1.667	0.581	0.004 *	−2.808 to −0.525	5.263
Income: SAR 15,001–20,000 vs. <5000	−1.839	0.581	0.002 *	−2.980 to −0.698	5.263
Income: SAR >20,000 vs. <5000	−0.947	0.603	0.117	−2.132 to 0.238	4.986
Age: 31–50 vs. 18–30	0.972	0.293	0.001 *	0.396 to 1.547	1.645
Age: >50 vs. 18–30	0.413	0.417	0.322	−0.406 to 1.232	1.747
Model Fit Statistics	*n* = 451; R^2^ = 0.179; adjusted R^2^ = 0.150; F(15, 435) = 6.314; model *p* < 0.001				
Practices of participants related to skin cancer					
Coastal region (coastal vs. non-coastal)	−0.282	0.217	0.194	−0.708 to 0.144	1.094
Gender (female vs. male)	0.589	0.209	0.005 *	0.178 to 1.001	1.114
Marital status (married vs. not married)	0.162	0.246	0.510	−0.321 to 0.645	1.299
Employment: student vs. unemployed	−0.299	0.423	0.479	−1.130 to 0.531	2.068
Employment: non-healthcare vs. unemployed	−0.673	0.305	0.028 *	−1.273 to −0.074	2.310
Employment: healthcare vs. unemployed	−0.593	0.330	0.073	−1.242 to 0.056	2.083
Education: below high school vs. university	0.563	0.652	0.388	−0.718 to 1.844	1.121
Education: high school/diploma vs. university	−0.258	0.264	0.328	−0.776 to 0.260	1.230
Education: higher education vs. university	0.409	0.317	0.199	−0.215 to 1.032	1.451
Income: SAR 5001–10,000 vs. <5000	−1.698	0.528	0.001 *	−2.735 to −0.661	3.585
Income: SAR 10,001–15,000 vs. <5000	−1.118	0.504	0.027 *	−2.109 to −0.126	5.263
Income: SAR 15,001–20,000 vs. <5000	−1.369	0.504	0.007 *	−2.360 to −0.377	5.263
Income: SAR >20,000 vs. <5000	−1.266	0.524	0.016 *	−2.296 to −0.237	4.986
Age: 31–50 vs. 18–30	0.247	0.254	0.333	−0.253 to 0.747	1.645
Age: >50 vs. 18–30	−0.180	0.362	0.618	−0.891 to 0.531	1.747
Model Fit Statistics	*n* = 451; R^2^ = 0.090; adjusted R^2^ = 0.059; F(15, 435) = 2.866; model *p* < 0.001				

* *p* < 0.05 indicates statistical significance. Reference categories: non-coastal region, unmarried status, unemployed status, university education, monthly income <SAR 5000, age of 18–30 years; male is the reference category for sex in the overall-sample models. B: unstandardized coefficient; SE: standard error; CI: confidence interval; VIF: variance inflation factor.

## Data Availability

The data presented in this study are available upon reasonable request from the corresponding author. The data are not publicly available due to privacy and confidentiality considerations related to the study participants.

## Supplementary Materials

The following supporting information can be downloaded at https://www.mdpi.com/article/10.3390/jcm15218570/s1, Table S1: Knowledge, attitude, and practice scores among female participants by sociodemographic characteristics. Table S2: Multivariable linear regression analyses among female participants.

## Abbreviations

The following abbreviations are used in this manuscript:

CI	Confidence interval
IQR	Interquartile range
SD	Standard deviation

## References

[1] Stratigos A., Garbe C., Lebbe C., Malvehy J., Del Marmol V., Pehamberger H., Peris K., Becker J.C., Zalaudek I., Saiag P. (2015). Diagnosis and treatment of invasive squamous cell carcinoma of the skin: European consensus-based interdisciplinary guideline. Eur. J. Cancer.

[2] Xiang F., Lucas R., Hales S., Neale R. (2014). Incidence of nonmelanoma skin cancer in relation to ambient UV radiation in white populations, 1978–2012: Empirical relationships. JAMA Dermatol..

[3] Das K., Cockerell C.J., Patil A., Pietkiewicz P., Giulini M., Grabbe S., Goldust M. (2021). Machine learning and its application in skin cancer. Int. J. Environ. Res. Public Health.

[4] Apalla Z., Nashan D., Weller R.B., Castellsagué X. (2017). Skin cancer: Epidemiology, disease burden, pathophysiology, diagnosis, and therapeutic approaches. Dermatol. Ther..

[5] Lee E., Karagenova R., Semenov Y.R. (2026). Melanoma prognostication using AI-guided histopathology. Int. J. Dermatol..

[6] Ciccarese G., Dalmasso B., Bruno W., Queirolo P., Pastorino L., Andreotti V., Spagnolo F., Tanda E., Ponti G., Massone C. (2020). Clinical, pathological and dermoscopic phenotype of MITF p.E318K carrier cutaneous melanoma patients. J. Transl. Med..

[7] Thomson J., Bewicke-Copley F., Anene C.A., Gulati A., Nagano A., Purdie K., Inman G.J., Proby C.M., Leigh I.M., Harwood C.A. (2021). The genomic landscape of actinic keratosis. J. Investig. Dermatol..

[8] Peris K., Alaibac M., Argenziano G., Di Stefani A., Fargnoli M.C., Frascione P., Gualdi G., Longo C., Moscarella E., Naldi L. (2018). Cutaneous squamous cell carcinoma. Italian guidelines by SIDeMaST adapted to and updating EADO/EDF/EORTC guidelines. G. Ital. Dermatol. Venereol..

[9] Saudi Health Council, National Cancer Center (2023). Cancer Incidence Report 2023: Saudi Arabia.

[10] Saudi Vision 2030 Saudi Vision 2030—Overview. https://www.vision2030.gov.sa/en/overview.

[11] Al Eqtisadiah Commercial Records of Women’s Salons in Saudi Arabia Increased by 83% Despite Increasing Operating Costs. Al Eqtisadiah, 13 March 2025. https://www.aleqt.com/2025/03/13/article_2758451.html.

[12] Lopes A., Pereira P., Oliveira H. (2025). Cytotoxic effects of a LED/UV nail polish dryer in human keratinocytes. Toxicol. In Vitro.

[13] Zhivagui M., Hoda A., Valenzuela N., Yeh Y.Y., Dai J., He Y., Nandi S.P., Otlu B., Van Houten B., Alexandrov L.B. (2023). DNA damage and somatic mutations in mammalian cells after irradiation with a nail polish dryer. Nat. Commun..

[14] Ordoñez T., Ruf M., Angles V., Brau G., Ferrario D., Mazzuoccolo L. (2024). Squamous cell carcinoma of the nail unit after repeated UV nail lamp exposure. A call for action?. An. Bras. Dermatol..

[15] Metko D., Mehta S., McMullen E., Bednar E.D., Abu-Hilal M. (2024). A systematic review of the risk of cutaneous malignancy associated with ultraviolet nail lamps: What is the price of beauty?. Eur. J. Dermatol..

[16] O’Sullivan N.A., Tait C.P. (2014). Tanning bed and nail lamp use and the risk of cutaneous malignancy: A review of the literature. Australas. J. Dermatol..

[17] Wehner M.R., Shive M.L., Chren M.M., Han J., Qureshi A.A., Linos E. (2012). Indoor tanning and non-melanoma skin cancer: Systematic review and meta-analysis. BMJ.

[18] Modenese A., Farnetani F., Andreoli A., Pellacani G., Gobba F. (2016). Questionnaire-based evaluation of occupational and non-occupational solar radiation exposure in a sample of Italian patients treated for actinic keratosis and other non-melanoma skin cancers. J. Eur. Acad. Dermatol. Venereol..

[19] Callens J., Van Eycken L., Henau K., Garmyn M. (2016). Epidemiology of basal and squamous cell carcinoma in Belgium: The need for a uniform and compulsory registration. J. Eur. Acad. Dermatol. Venereol..

[20] Marks R., Rennie G., Selwood T. (1988). The relationship of basal cell carcinomas and squamous cell carcinomas to solar keratoses. Arch. Dermatol..

[21] Bin Dayel S. (2025). Skin cancer awareness, attitude, and sun protection behavior among medical students in Saudi Arabia: A cross-sectional study. Medicine.

[22] Ali M., Narapureddy B.R. (2026). Assessment of knowledge, attitudes, and practices regarding sun exposure and sun protection among female students living in high-altitude areas, Abha, Saudi Arabia: A cross-sectional study. PeerJ.

[23] Bahashwan E. (2024). Awareness and knowledge of sun exposure and use of sunscreen among adults in Aseer region, Saudi Arabia. Saudi Pharm. J..

[24] Baghani M., Robati R.M., Mozafari N., Baghani M., Kassir M., Sheibani F., Mansouri V. (2024). Knowledge, attitude, and practice toward skin cancer among patients of dermatology clinics and medical students/general practitioners. J. Skin Cancer.

[25] Assiri A., Hamzi S.A., Hamzi Y.A., Muyidi A.Y., Harthi F.M., Qaysi M.M., Jadah A.A., Safhi A.M., Hobani A.H. (2024). Knowledge, attitude, reception, and preventive practices towards skin photoaging among the general population in Jazan, Saudi Arabia. Cureus.

