# Supplementary material for: Public Knowledge, Attitudes, and Practices Regarding Skin Cancer, Risk Factors, and Preventive Measures in Saudi Arabia: A Cross-Sectional Study
Source: J Clin Med. 2026 Nov 3;15(21):8570. doi: 10.3390/jcm15218570 (PMC13764340; doi:10.3390/jcm15218570)
Supplement: Supplementary file 1 [file jcm-15-08570-s001.zip › jcm-4576010-supplementary.pdf]

Supplementary Table S1. Knowledge, Attitude, and Practice Scores Among Female Participants by Sociodemographic Characteristics.

Variable	Category	Knowledge score/26	Attitude score/12	Practice score/20
Coastal region	Non-Coastal	13.62 ± 4.52<br>13.00 (11.00-16.00)	6.24 ± 2.72<br>6.00 (4.00-7.00)	6.79 ± 2.79<br>6.00 (5.00-8.00)
	Coastal	14.14 ± 5.24<br>14.00 (10.00-18.25)	7.17 ± 3.20<br>6.00 (4.75-10.00)	6.42 ± 2.75<br>6.00 (5.00-8.00)
	p-value	0.669	0.042*	0.392
	Effect size	0.029	0.138	0.058
Age (years)	18-30	13.57 ± 3.97<br>14.00 (11.00-16.00)	5.47 ± 2.18<br>6.00 (4.00-6.00)	6.14 ± 2.52<br>6.00 (5.00-7.00)
	31-50	13.59 ± 4.98<br>13.00 (10.00-16.25)	7.19 ± 3.20<br>7.00 (5.00-10.00)	6.79 ± 2.90<br>6.00 (5.00-8.00)
	>50	15.56 ± 5.98<br>16.00 (12.50-19.50)	7.24 ± 2.76<br>7.00 (5.00-10.00)	8.00 ± 2.65<br>8.00 (6.00-9.00)
	p-value	0.138	<0.001*	0.010*
	Effect size	0.009	0.065	0.033
Marital status	Not married	14.07 ± 4.28<br>14.00 (11.00-16.00)	6.40 ± 2.79<br>6.00 (4.00-8.00)	6.51 ± 3.04<br>6.00 (5.00-8.00)
	Married	13.67 ± 5.04<br>13.00 (10.00-17.00)	6.66 ± 3.01<br>6.00 (4.00-10.00)	6.74 ± 2.63<br>6.00 (5.00-8.00)
	p-value	0.278	0.674	0.340
	Effect size	0.073	0.028	0.065
Education level	<High school	12.86 ± 5.21<br>15.00 (6.00-17.00)	7.43 ± 2.23<br>7.00 (5.00-10.00)	8.43 ± 3.60<br>7.00 (6.00-9.00)
	High school diploma	12.90 ± 3.39<br>13.00 (10.00-15.00)	5.49 ± 2.30<br>5.00 (4.00-7.00)	5.90 ± 2.64<br>6.00 (4.00-7.00)
	University	13.20 ± 4.83<br>13.00 (10.00-16.00)	6.42 ± 2.83<br>6.00 (4.00-8.00)	6.60 ± 2.71<br>6.00 (5.00-8.00)
	Higher education	16.39 ± 4.86<br>16.00 (12.00-20.00)	7.78 ± 3.36<br>8.00 (5.00-11.00)	7.20 ± 2.78<br>7.00 (5.00-10.00)
	p-value	0.004*	0.004*	0.032*
	Effect size	0.049	0.047	0.027
Employment status	Unemployed	12.81 ± 6.36<br>11.50 (7.75-17.25)	7.35 ± 2.90<br>7.00 (5.00-10.00)	7.81 ± 3.34<br>8.00 (5.00-9.00)
	Student	14.84 ± 4.24<br>16.00 (12.00-17.50)	5.80 ± 2.83<br>6.00 (4.00-7.00)	6.16 ± 2.91<br>6.00 (4.00-7.00)

Variable	Category	Knowledge score/26	Attitude score/12	Practice score/20
	Employed in the non-health sector	14.06 ± 4.10<br>13.50 (11.00-16.75)	6.91 ± 3.14<br>6.00 (5.00-10.00)	6.50 ± 2.73<br>6.00 (5.00-8.00)
	Employed in the health sector	13.92 ± 4.12<br>13.00 (11.00-16.00)	5.55 ± 2.27<br>6.00 (4.00-6.00)	5.96 ± 1.56<br>6.00 (5.00-7.00)
	p-value	0.175	0.004*	0.005*
	Effect size	0.009	0.047	0.047
Monthly income	≤SR 5,000	13.25 ± 5.27<br>14.00 (10.25-16.00)	8.00 ± 3.08<br>8.50 (5.00-10.00)	8.75 ± 2.54<br>8.00 (8.00-9.00)
	SR 5,001-10,000	13.97 ± 4.08<br>14.00 (11.00-16.00)	6.26 ± 2.96<br>6.00 (4.00-9.00)	5.82 ± 2.56<br>6.00 (4.00-7.00)
	SR 10,001-15,000	13.41 ± 4.53<br>13.50 (10.00-16.25)	6.03 ± 2.78<br>6.00 (4.00-8.00)	6.22 ± 2.58<br>6.00 (4.00-7.25)
	SR 15,001-20,000	12.43 ± 4.23<br>12.00 (10.00-15.00)	5.92 ± 2.26<br>6.00 (4.00-7.00)	6.44 ± 2.72<br>6.00 (5.00-7.00)
	>SR 20,000	16.21 ± 5.36<br>16.00 (12.00-21.00)	7.85 ± 3.36<br>8.00 (5.00-11.00)	7.38 ± 2.89<br>7.00 (5.00-10.00)
	p-value	0.007*	0.004*	<0.001*
	Effect size	0.048	0.052	0.075

* $p < 0.05$ indicates statistical significance. ($n = 218$). IQR: Interquartile range; SD: Standard deviation; SR: Saudi Riyal.

Supplementary Table S2. Multivariable linear regression analyses among female participants.

Independent Variable/Comparison	B	SE	*p*-value	95% CI for B	VIF
Knowledge of females about skin cancer					
Coastal region (coastal vs non-coastal)	0.382	0.685	0.578	-0.969 to 1.733	1.139
Marital status (married vs not married)	-0.447	0.762	0.558	-1.949 to 1.055	1.382
Employment: student vs unemployed	2.386	1.273	0.062	-0.124 to 4.896	1.736
Employment: non-healthcare vs unemployed	0.498	0.899	0.580	-1.275 to 2.270	2.053
Employment: healthcare vs unemployed	1.531	0.966	0.115	-0.375 to 3.437	1.766
Education: below high school vs university	-1.089	1.921	0.571	-4.878 to 2.699	1.211
Education: high school/diploma vs university	-0.719	0.902	0.426	-2.498 to 1.060	1.262
Education: higher education vs university	2.533	0.999	0.012*	0.563 to 4.503	1.754
Income: SAR 5,001–10,000 vs <5,000	0.153	1.456	0.916	-2.718 to 3.025	2.946
Income: SAR 10,001–15,000 vs <5,000	-0.620	1.409	0.660	-3.399 to 2.159	4.093

Independent Variable/Comparison	B	SE	*p*-value	95% CI for B	VIF
Income: SAR 15,001–20,000 vs <5,000	-1.378	1.402	0.327	-4.143 to 1.386	4.261
Income: SAR >20,000 vs <5,000	1.116	1.521	0.464	-1.884 to 4.116	4.131
Age: 31–50 vs 18–30	-0.254	0.784	0.746	-1.800 to 1.291	1.617
Age: >50 vs 18–30	1.751	1.197	0.145	-0.610 to 4.111	1.535
Model Fit Statistics	n = 218; R^2 = 0.156; adjusted R^2 = 0.098; F(14, 203) = 2.689; model p < 0.001				
Attitude of females toward skin cancer					
Coastal region (coastal vs non-coastal)	0.768	0.413	0.064	-0.045 to 1.581	1.139
Marital status (married vs not married)	-0.643	0.459	0.163	-1.547 to 0.261	1.382
Employment: student vs unemployed	-0.566	0.766	0.461	-2.077 to 0.945	1.736
Employment: non-healthcare vs unemployed	-0.575	0.541	0.290	-1.642 to 0.492	2.053
Employment: healthcare vs unemployed	-1.261	0.582	0.031*	-2.408 to -0.113	1.766
Education: below high school vs university	0.732	1.157	0.527	-1.548 to 3.013	1.211
Education: high school/diploma vs university	-0.432	0.543	0.427	-1.503 to 0.639	1.262
Education: higher education vs university	0.943	0.601	0.119	-0.243 to 2.129	1.754
Income: SAR 5,001–10,000 vs <5,000	-0.850	0.877	0.333	-2.578 to 0.878	2.946
Income: SAR 10,001–15,000 vs <5,000	-1.250	0.848	0.142	-2.923 to 0.423	4.093
Income: SAR 15,001–20,000 vs <5,000	-1.230	0.844	0.147	-2.894 to 0.434	4.261
Income: SAR >20,000 vs <5,000	-0.371	0.916	0.686	-2.177 to 1.435	4.131
Age: 31–50 vs 18–30	1.271	0.472	0.008*	0.341 to 2.201	1.617
Age: >50 vs 18–30	0.928	0.721	0.199	-0.493 to 2.349	1.535
Model Fit Statistics	n = 218; R^2 = 0.184; adjusted R^2 = 0.128; F(14, 203) = 3.280; model p < 0.001				
Practice of females related to skin cancer					
Coastal region (coastal vs non-coastal)	-0.587	0.400	0.144	-1.376 to 0.202	1.139
Marital status (married vs not married)	-0.358	0.445	0.423	-1.235 to 0.520	1.382
Employment: student vs unemployed	-1.364	0.743	0.068	-2.830 to 0.102	1.736
Employment: non-healthcare vs unemployed	-1.380	0.525	0.009*	-2.415 to -0.345	2.053
Employment: healthcare vs unemployed	-1.487	0.564	0.009*	-2.600 to -0.374	1.766
Education: below high school vs university	0.847	1.122	0.451	-1.366 to 3.060	1.211
Education: high school/diploma vs university	-0.444	0.527	0.401	-1.483 to 0.596	1.262
Education: higher education vs university	0.601	0.584	0.304	-0.550 to 1.752	1.754
Income: SAR 5,001–10,000 vs <5,000	-2.007	0.851	0.019*	-3.684 to -0.330	2.946
Income: SAR 10,001–15,000 vs <5,000	-1.514	0.823	0.067	-3.137 to 0.109	4.093

Independent Variable/Comparison	B	SE	*p*-value	95% CI for B	VIF
Income: SAR 15,001–20,000 vs <5,000	-1.468	0.819	0.074	-3.083 to 0.146	4.261
Income: SAR >20,000 vs <5,000	-0.809	0.889	0.364	-2.561 to 0.944	4.131
Age: 31–50 vs 18–30	0.135	0.458	0.768	-0.767 to 1.038	1.617
Age: >50 vs 18–30	0.501	0.699	0.475	-0.878 to 1.879	1.535
Model Fit Statistics	n = 218; R^2 = 0.143; adjusted R^2 = 0.084; F(14, 203) = 2.413; model p < 0.004				

* $p < 0.05$ indicates statistical significance. (n = 218). Reference categories: non-coastal region, unmarried status, unemployed status, university education, monthly income <SAR 5,000, age of 18–30 years. B: unstandardized coefficient; SE: standard error; CI: confidence interval; VIF: variance inflation factor.
